# Comparison of Intraocular Lens Stability and Reversibility in Phacovitrectomy With Air or Gas Tamponade Using One- and Three-Piece Intraocular Lenses

**DOI:** 10.7759/cureus.87252

**Published:** 2025-07-03

**Authors:** Yuji Yoshikawa, Jun Makita, Kei Shinoda

**Affiliations:** 1 Ophthalmology, Saitama Medical University Hospital, Saitama, JPN

**Keywords:** anterior chamber depth, gas tamponade, intraocular lens displacement, intraocular lens position, phacovitrectomy

## Abstract

Purpose

To compare intraocular lens (IOL) stability and reversibility in phacovitrectomy with air or gas tamponade between one- and three-piece intraocular lenses (IOLs).

Study design

This retrospective cross-sectional study was conducted at Saitama Medical University Hospital from January to July 2023.

Methods

This study involved 19 patients who underwent phacovitrectomy with either air or sulfur hexafluoride gas tamponade and postoperative anterior segment optical coherence tomography. Patients who underwent NSP-3 (NIDEK Co., Ltd., Japan) or NX70s (Santen Pharmaceutical Co., Japan) implantation were evaluated. The displacement force (mN) of the IOL was evaluated in a verification experiment using the NSP-2 (NIDEK Co., Ltd., Japan) and the NX70s. The NSP-2 and NSP-3 share the same lens platform.

Results

The median age was 66 (58, 77) [median (quartiles)] years for NX70s (n=8) and 67 (55, 71) years for NSP-3 (n=11). The anterior chamber depth (ACD) and IOL position values were substantially elevated after the air or gas disappeared for both IOLs. Comparing the IOLs for the ACD and IOL positions at each time point, no significant difference was found in the ACD and IOL position values in 100% gas or air.

However, ACD and IOL position values of the NSP-3 were markedly higher than those of the NX70s in 0% gas or air. In the verification experiment, NSP-2 required a higher IOL displacement force than the NX70s. Following 48 h of 0.5 mm push, the percentage change in the axial distance was smaller for NSP-2 (−6.9%) than for NX70s (−15.5%).

Conclusion

NSP3 was as stable as NX70s in phacovitrectomy with tamponade and demonstrated better reversibility than NX70s.

## Introduction

While various formula improvements have improved the precision of postoperative refractive outcomes in standalone cataract surgery, challenges persist. Moreover, managing postoperative refractive errors following phacovitrectomy remains more complicated than in cataract surgery alone [1-5]. It has been reported that single-piece lenses are more stable than three-piece lenses in cataract surgery alone [6, 7].On the other hand, it has been reported that the effective lens position changes by vitreous surgery, especially with gas tamponade [8].

In particular, the use of gas tamponade during vitrectomy results in a shallower postoperative anterior chamber depth (ACD) compared to procedures without tamponade [9]. One contributing factor is the z-axis stability of the intraocular lens (IOL) in the presence of gas tamponade. Additionally, IOL stability varies depending on the type of lens, such as one-piece, three-piece, and lenses with 6 mm or 7 mm optics [10, 11]. A comparative study of different types of three-piece IOLs reported that when gas tamponade was used, the 7.0 mm diameter IOL moved less than the 6.5 mm diameter IOL [12].

This study aimed to clinically and experimentally evaluate the stability and reversibility of one-piece, 6mm IOL and three-piece, 7mm IOL during phacovitrectomy with air or gas tamponade. A portion of the data in this article (NX70s group) is available as a preprint [13]. A portion of this article was presented as a poster at the 2025 Fuji Retina conference (Poster: Yoshikawa Y, Nakajima K, Sakai E, Nagasaka S, Shinoda K. IOL Position Stability After Phacovitrectomy With Air or Gas Tamponade Between NSP-3 and NX70s. March 28-30, 2025).

## Materials and methods

This retrospective cross-sectional study was approved by the Ethics Committee of Saitama Medical University in Iruma, Japan (No. 2023-095). The study was conducted in accordance with the principles of the Declaration of Helsinki, and informed consent was obtained from all participants.

Subjects

The inclusion criteria comprised patients who underwent phacovitrectomy with air or sulfur hexafluoride (SF₆) gas tamponade at Saitama Medical University Hospital between January and July 2023. Patients who received anterior segment optical coherence tomography (AS-OCT) evaluations using CASIA2 (TOMEY, Inc. Japan), both on the first day after surgery and after the complete absorption of air or gas. Patients were implanted with either the NSP-3 (NIDEK Co., Ltd., Japan) or NX70s (Santen Pharmaceutical Co., Japan) IOLs.

Patients with incomplete continuous curvilinear capsulorhexis (CCC) during surgery or those who experienced posterior capsule rupture were excluded from the study.

Surgery

A retinal specialist conducted cataract surgery through a 2.4-mm corneal incision, concurrently performing a 25- or 27-gauge pars plana vitrectomy. The CCC was carried out to ensure complete coverage of the IOL optics. Trocars were inserted into the vitreous cavity at a 30° angle relative to the scleral surface, positioned 3.5 mm posterior to the corneal limbus. All patients underwent peripheral vitreous shaving followed by a fluid-air exchange. Based on intraoperative findings, the vitreous cavity was replaced with 20% SF₆ gas when necessary. Each procedure was completed with IOL implantation, and the vitreous cavity filled with either air or 20% SF₆.

Verification experiment

The displacement force of the IOL was measured according to previously established methods [10, 11, 13]. The IOL power used was +21.0 diopters. In this experiment, the NSP-2 lens (NIDEK Co., LTD, Japan) was employed instead of the NSP-3. Although NSP-2 differs from NSP-3 in terms of the presence or absence of higher-order aberrations in the optical zone, their structural designs are identical. Measurements were conducted in air at room temperature (31°C), the maximum temperature that can be controlled by an air conditioner.

**Figure 1:**
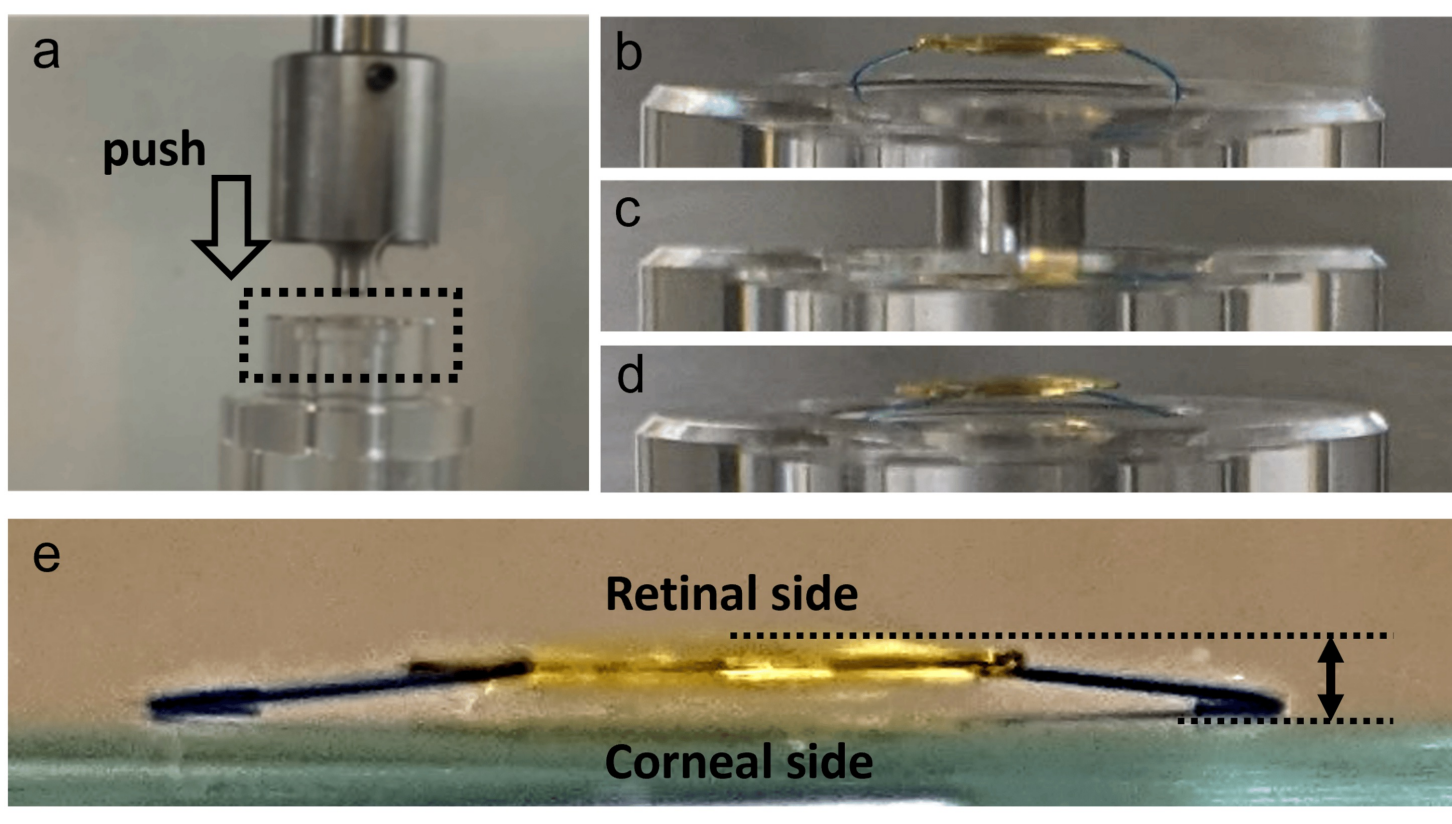
Verification experiment for evaluation of displacement force and reversibility of intraocular lenses (IOLs) (a) The displacement force was measured by applying pressure with a digital micrometer head onto the retinal side of the IOL. The IOL was gradually pushed forward in 0.1 mm increments up to a total displacement of 0.5 mm. At each 0.1 mm step, the force needed to move the IOL was recorded in millinewtons (mN) using an electronic balance; (b) IOL in a holder; (c) IOL pushed in from the retinal side; (d) IOL released from the loading; (e) The axial distance is defined as the distance from the posterior surface of the optics (retinal side) to the anterior edge of the haptics (corneal side).

To replicate the displacement force applied when the IOL is pushed from the retinal side by air or gas, the IOL was secured in a holder with a 10 mm diameter, and a digital micrometer head (3.0 mm diameter; Mitutoyo, Kawasaki, Japan) was used to apply force from the retinal side toward the corneal side. The displacement force (mN) was recorded using an electronic balance (SHIMADZU, Kyoto, Japan). The IOL was pushed up to 0.5 mm toward the corneal side, and displacement force measurements were taken every 0.1 mm (Figures 1a, 1b, 1c). To assess the reversibility of the shape of IOL haptics, the change in the z-axis distance between the optics and haptics was measured before and 48 hours after the IOL was displaced. The z-axis distance measurements were performed after removing the IOL from the holder. The axial distance is defined as the distance from the posterior surface of the optics (retinal side) to the anterior edge of the haptics (corneal side) (Figures 1d, 1e). One lens each of the NSP-2 and NX70s was used for the push-in and recovery experiments, respectively. Each experiment was conducted only once.

Data analysis

ACD measurements were taken using the CASIA 2 system. Measurements were taken twice, once when the vitreous cavity was 100% filled with air or SF₆ gas and once when the air or gas was completely absorbed. IOL position was calculated as the ratio of the change in ACD before and after surgery to preoperative lens thickness, defined as (postoperative ACD - preoperative ACD)/pre operative lens thickness, according to a previously published method [10].

The postoperative refractive error was defined as the difference between the predicted refraction obtained from the Barrett Universal II formula using the CASIA 2 and OA-2000 (TOMEY, Inc. Japan), and the subjective refraction after gas absorption.

All data, including ACD, IOL position values, and postoperative refractive error, were expressed as medians with interquartile ranges. ACDs and IOL positions were compared using the Wilcoxon signed-rank test for temporal variation. Differences between the two IOL groups at each time point were also analyzed using the Mann-Whitney U test. The postoperative refractive errors of the two IOLs were compared using the Mann-Whitney U test.

To evaluate IOL reversibility, changes in the distance in the z-direction between the optics and the haptics of the IOL were compared before and after 48 hours of compression. The ratio of change was calculated as (distance after compression - distance before compression)/distance before compression.

Statistical analyses were conducted using JMP 10.1 software (SAS Institute, Cary, NC, USA). A P-value of less than 0.05 was regarded as statistically significant

## Results

No subjects met the exclusion criteria, and finally, 19 subjects were eligible in this analysis.

The median patient age was 66 (58, 77) years [median (quartiles)] for the NX70s (n = 8) and 67 (55, 71) years for the NSP-3 (n = 11); No significant difference was observed between the median ages of the groups. Additionally, no significant differences were observed in sex, axial length, lens thickness, preoperative ACD, ratio of air to SF₆, or time to CASIA2 examination (TOMEY, Inc., Japan). The postoperative refractive error was -0.50 (-0.88, -0.02) in the NSP-3 group and 0.13 (-0.45, 0.45) in the NX70s group, with no statistically significant difference between the two groups (Table 1).

**Table 1:** Group characteristics ACD: anterior chamber depth; SF₆: sulfur hexafluoride; MH: macular hole; RRD: rhegmatogenous retinal detachment; PDR: proliferative diabetic retinopathy; VMTS: vitreomacular traction syndrome. *Mann–Whitney U test †Fisher's exact test.

	NX70s (n=8)	NSP-3 (n=11)	p-value
Age, years	66 (58, 77)	67 (55, 71)	0.456*
Sex (female), n	6 (75%)	5 (45%)	0.352^†^
(male), n	2 (25%)	6 (55%)	
Axial length, mm	23.6 (23.2, 24.5)	25,3 (23.6, 25.9)	0.186*
Lens thickness, mm	4.5 (4.2, 4.9)	4.5 (4.1, 4.9)	0.869*
Preoperative ACD, mm	2.59 (2.36, 2.74)	2.75 (2.36, 2.94)	0.385*
Postoperative refractive error, diopter	0.13 (-0.45, 0.45)	-0.50 (-0.88, -0.02)	0.107*
Tamponade (Air), n	2 (25%)	2 (18%)	1.000^†^
(SF₆), n	6 (75%)	9 (82%)	
Time to examination (0%), days	30 (14, 43)	20 (19, 32)	0.772*
Disease type, n			
MH	6 (75%)	3 (27%)	
RRD	2 (25%)	6 (55%)	
PDR	0 (0%)	1 (9%)	
VMTS	0 (0%)	1 (9%)	

**Figure 2:**
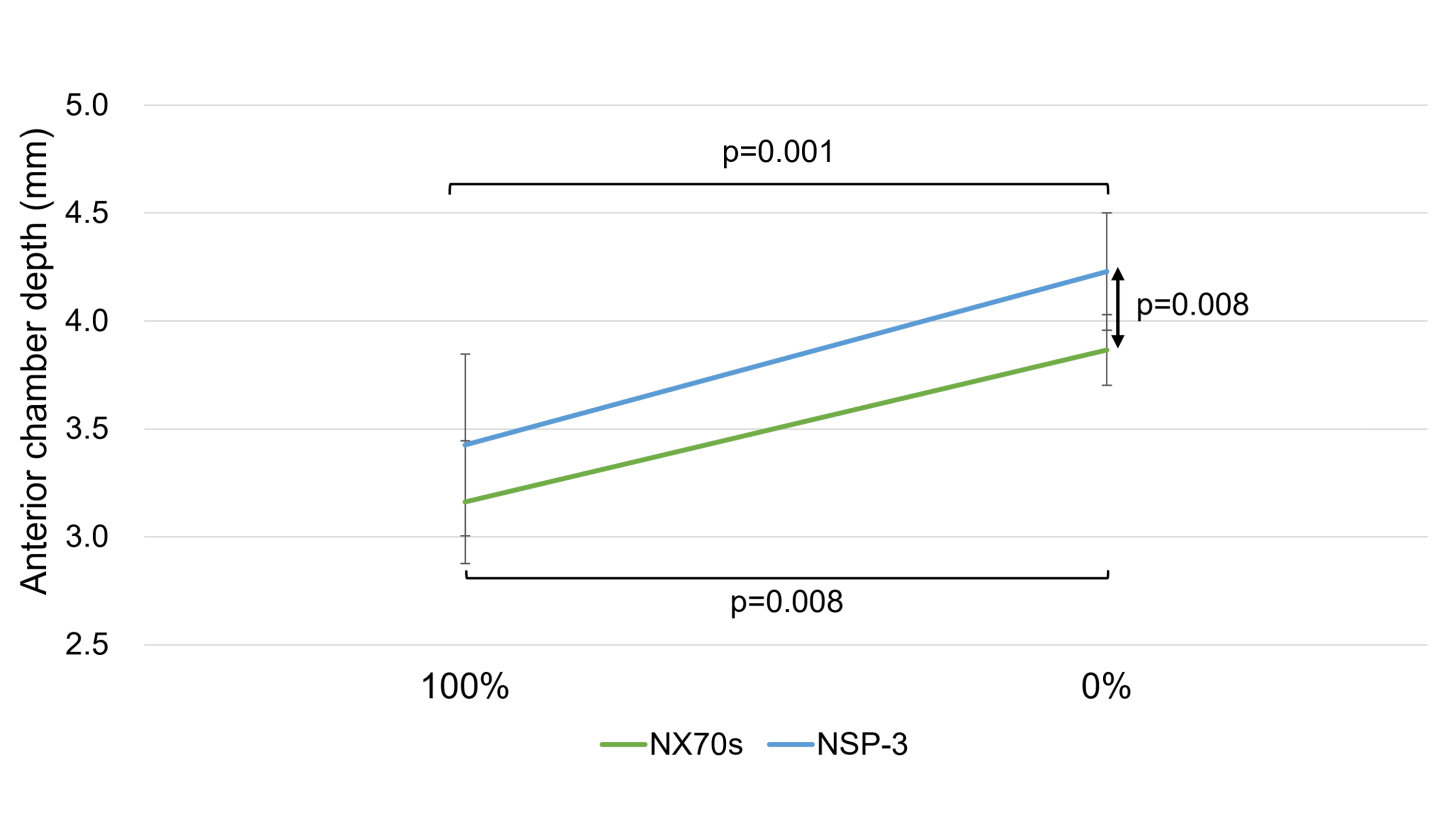
Change in anterior chamber depth (ACD) This figure shows the ACD changes at 100% and 0% gas or air conditions for both NSP-3 and NX70s lenses. For both intraocular lens (IOL) types, the ACD values significantly increased after the complete disappearance of air or gas.When comparing the two IOLs at each measurement point, no significant difference was found under the 100% gas or air condition. However, at 0% gas or air, the ACD values for NSP-3 were significantly greater than those for NX70s (p = 0.008).

**Figure 3:**
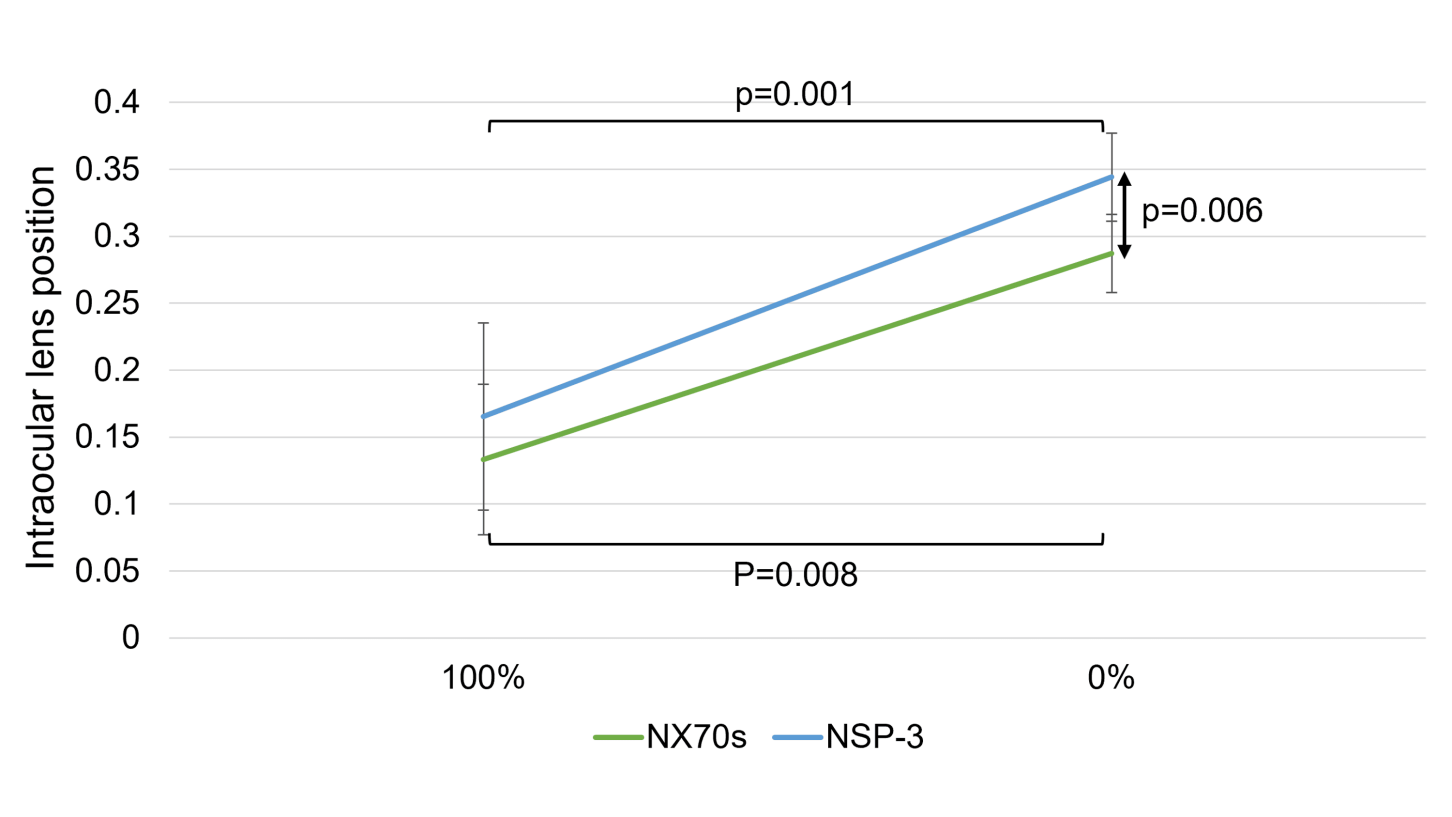
Change in the intraocular lens (IOL) position This figure shows the changes in IOL position for NSP-3 and NX70s at 100% and 0% gas or air. Both IOLs exhibited a notable increase in position after the air or gas had been completely absorbed. When comparing the two IOLs at each time point, no significant difference was found under 100% gas or air conditions. However, at 0%, the IOL position values for NSP-3 were significantly greater than those for NX70s (p = 0.006).

The values of the ACD and IOL position significantly increased after the air or gas disappeared from both IOLs. Furthermore, no significant difference was observed in the ACD and IOL position values between both IOLs at 100% gas or air. At 0% tamponade, the ACD and IOL position values for the NSP-3 were significantly greater than those for the NX70s (ACD: 4.31 [4.06, 4.41] mm for NSP-3 vs. 3.92 [3.70, 4.02] mm for NX70s, p = 0.008; IOL position: 0.35 [0.32, 0.36] for NSP-3 vs. 0.29 [0.26, 0.31] for NX70s, p = 0.006) (Figures 2, 3).

**Figure 4:**
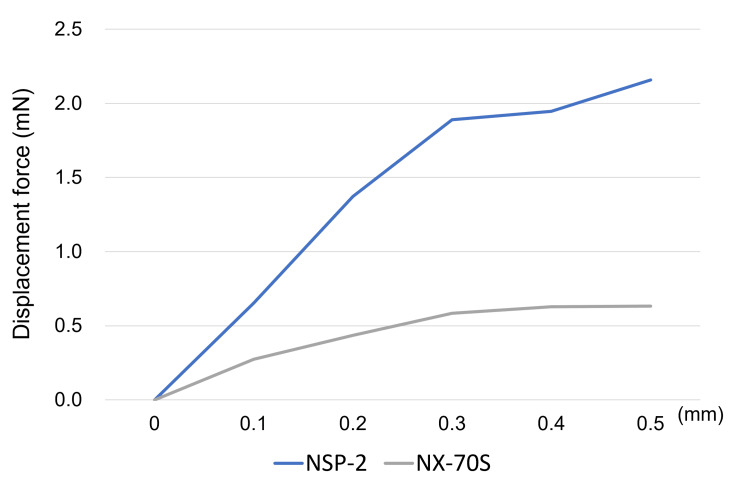
Displacement force This figure depicts the displacement force needed to move the intraocular lenses (IOLs). For both NSP-2 and NX70s, the force required to displace the lens increased with the extent of movement. Nevertheless, the NSP-2 demanded a higher displacement force than the NX70s to shift the optics from the retinal side toward the corneal side.

In the verification experiment, the NSP-2 required a higher displacement force to resist pushing from the retinal side compared to the NX70s (displacement force at 0.5 mm: NSP-2, 2.16 mN; NX70s, 0.64 mN) (Figure 4).

**Figure 5:**
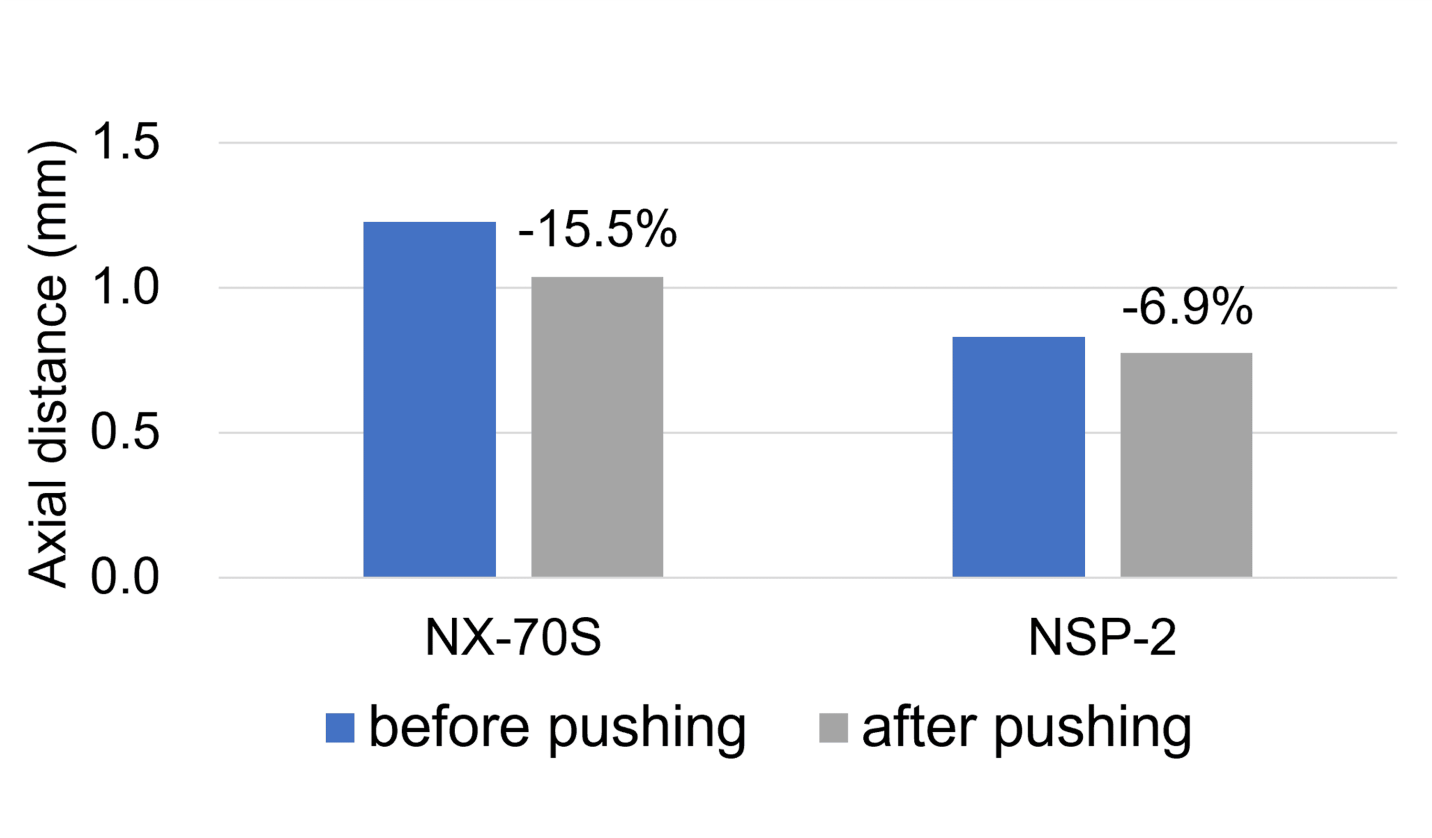
Changes in the axial distance of intraocular lens (IOL) The graph illustrates the changes in axial distance for each IOL before applying a 0.5 mm compression (at 0 hours) and after 48 hours of sustained compression. Compared to NX70s, which showed a 15.5% reduction, the NSP-2 exhibited a smaller decrease of 6.9% in axial distance.

The axial distances before applying a 0.5 mm displacement (at 0 hours) were 0.831 mm for NSP-2 and 1.228 mm for NX70s. The axial distances were 0.774 mm for NSP-2 and 1.038 mm for NX70s after 48 h of the 0.5 mm push. The percentage change in the axial distance was smaller for NSP-2 (-6.9%) than for NX70s (-15.5%) (Figure 5).

## Discussion

This study assessed the z-axis stability and reversibility of NSP-3 and NX70s under two clinical conditions (phacovitrectomy with air or SF₆ gas tamponade) and performed verification experiments.

In general, three-piece IOLs and large-diameter IOLs are preferred for simultaneous vitrectomy and cataract surgery because of the stability of the IOL and the ease of viewing the fundus through the IOL [14, 15]. However, attempts have recently been made to implant multifocal IOLs for vitrectomy, increasing the requirement for a one-piece IOL in phacovitrectomy [16, 17]. Although most one-piece IOLs have a diameter of 6.0 mm and their lens positioning may be influenced by gas tamponade [8], the NS60YG (NIDEK Co., LTD, Japan), a one-piece IOL, demonstrated greater stability compared to the 7 mm, three-piece X70 lens [10]. Interestingly, the stability varied among different one-piece IOLs, especially NS60YG, YP2.2, and ZCB00V need higher displacement force than that of SN60WF, XY-1, and 255 [11]. In the NSP-3 and NX70s, the IOL position increased and shifted toward the retina with a decrease in the gas or air. This indicated that the IOL was pushed toward the cornea by air or gas in the vitreous cavity. Thus, the IOL shifted toward the retina when the air or gas disappeared.

Earlier research has indicated that the IOL position after cataract surgery alone is approximately 0.35 to 0.36 [9]. In this study, after the air or gas tamponade had dissipated, the IOL position for the NSP-3 was 0.35 (0.32, 0.36), suggesting that the lens returned to a position comparable to that observed in cataract surgery alone. In contrast, the NX70s remained more anteriorly displaced toward the cornea than in cataract surgery cases, even after the air or gas had disappeared, showing a value of 0.29 (0.26, 0.31).

Furthermore, the axial distance between the optics and haptics before pushing was greater for the NX70s than for the NSP-3. (Figure 5) This is because the NX70s is a three-piece lens, and the optics and haptics are angled. Normally, the NX70s would set back more toward the retina than the NSP-3 when the lens is placed in the capsular bag owing to this structural feature; therefore, the ACD and IOL positions should be larger than those of the NSP-3. However, it is an important finding that the value of IOL position after air or gas disappeared was greater in NSP-3. In the validation experiment (Figure 5), 48 h of pushing caused deformation of the IOL structure and an alteration in the positional relationship between the optics and haptics. Particularly, the deformation of NX70s (-15.5%) was greater than that of NSP-3 (-6.9%). This suggests that air or gas temporarily shifted the IOL toward the cornea, causing deformation; consequently, the position of the optics remained fixed anteriorly in the NX70s, even after the air or gas disappeared. The shift and deformation of the IOL due to gas tamponade shown in this study may be one of the causes of myopia due to gas tamponade shown in previous studies [9, 18].

Although this study did not identify specific factors influencing the stability and reversibility of IOL position, previous research has reported that several factors affect IOL stability, such as the area of the haptic junction [11], the number of haptics, and their design [19]. Furthermore, the shape and composition of the haptics may also play a role in IOL reversibility. While there was a significant difference in postoperative ACD and IOL position, no significant difference was found in postoperative refractive error between two IOLs in this study. One possible reason for this is the lack of personalized A-constant correction for each IOLs, as well as potential differences among surgeons, which may have influenced the postoperative refraction. A prospective study that personalizes the A-constant for each IOLs and limits the number of surgeons would be necessary to more accurately evaluate postoperative refractive errors in the future.

This study has several limitations, including a small sample size, limited statistical power, its retrospective design, involvement of multiple surgeons, possible selection bias in IOL assignment, and the use of two different tamponade agents. Another limitation of this study is that the verification experiment does not perfectly replicate the intraocular environment and clinical condition. However, all surgeons provided complete CCC, which covered IOL optics, and the ratio of SF₆ gas tamponade was comparable between NSP-3 and NX70s. The peripapillary vitreous shaving varies with the disease. Careful vitreous shaving of the most peripheral retina may result in fluctuations in lens position in response to changes in anterior chamber pressure during surgery due to the weakening of the Zinn ligament [20]. In the case of air or gas tamponade, the IOL may be more likely to shift toward the cornea side due to the weakening of the Zinn ligament. In this study, even though diseases that would require careful vitreous shaving of the most peripheral retina, such as rhegmatogenous retinal detachment (RRD) and proliferative diabetic retinopathy (PDR), were more common in NSP-3, the ACD and IOL position value at 100% air or SF₆ gas was similar to NX70s.

In this study, the time to anterior segment examination at 0% air or gas was approximately 20-30 days after surgery. On the other hand, the experimental data validating IOL reversibility evaluated the recovery of lens shape immediately after the release of pressure at 48 hours. As a result, the impact of air or gas compression beyond 48 hours on the IOL could not be assessed. In particular, RRD and PDR are more likely to involve long-term retention of gas compared to other diseases. Although no significant difference was observed in this experiment, the proportion of eyes filled with SF₆ gas was higher in the NSP-3 group than in the NX70s group. While a longer duration of gas tamponade may affect the deformation of the IOL, our results suggested less IOL deformation in the NSP-3 group, which contained a higher proportion of SF₆ gas. Therefore, it is considered that differences in underlying diseases and the proportion of SF₆ gas had little impact on this result.

In addition, although the duration of postoperative positioning and time spent in the supine position may differ depending on the underlying disease, these factors were not accounted for in this study. Future prospective studies that unify the type of gas used and postoperative positional restrictions, as well as underlying diseases, will be necessary.

## Conclusions

NSP3 showed stability comparable to NX70s and better reversibility than NX70s in vitrectomy with tamponade; the IOL structures of NSP-3 and NSP-2 are considered suitable for phacovitrectomy, despite being one-piece and 6mm structures.

## References

[1] Retzlaff JA, Sanders DR, Kraff MC (1990). Development of the SRK/T intraocular lens implant power calculation formula. J Cataract Refract Surg.

[2] Holladay JT, Prager TC, Chandler TY, Musgrove KH, Lewis JW, Ruiz RS (1988). A three-part system for refining intraocular lens power calculations. J Cataract Refract Surg.

[3] Olsen T (2006). Prediction of the effective postoperative (intraocular lens) anterior chamber depth. J Cataract Refract Surg.

[4] Barrett GD (1993). An improved universal theoretical formula for intraocular lens power prediction. J Cataract Refract Surg.

[5] Ghoreyshi M, Khalilian A, Peyman M, Mohammadinia M, Peyman A (2018). Comparison of OKULIX ray-tracing software with SRK-T and Hoffer-Q formula in intraocular lens power calculation. J Curr Ophthalmol.

[6] Behrouz MJ, Kheirkhah A, Hashemian H, Nazari R (2010). Anterior segment parameters: comparison of 1-piece and 3-piece acrylic foldable intraocular lenses. J Cataract Refract Surg.

[7] Zhong X, Long E, Chen W (2016). Comparisons of the in-the-bag stabilities of single-piece and three-piece intraocular lenses for age-related cataract patients: a randomized controlled trial. BMC Ophthalmol.

[8] Debourdeau E, Pineau P, Chamard C (2025). Clinical and biometric factors associated with prediction errors related to lens position in vitrectomized patients. Ophthalmic Res.

[9] Shiraki N, Wakabayashi T, Sakaguchi H, Nishida K (2020). Effect of gas tamponade on the intraocular lens position and refractive error after phacovitrectomy: a swept-source anterior segment oct analysis. Ophthalmology.

[10] Akiyama A, Yokota H, Aso H, Hanazaki H, Iwasaki M, Yamagami S, Nagaoka T (2022). Comparison of postoperative stability of intraocular lenses after phacovitrectomy for rhegmatogenous retinal detachment. J Clin Med.

[11] Mochiji M, Kaidzu S, Ishiba Y, Matsuda Y, Tanito M (2020). Measurement of force required for anterior displacement of intraocular lenses and its defining parameters. Materials (Basel).

[12] Watanabe A, Shibata T, Ozaki M, Okano K, Kozaki K, Tsuneoka H (2010). Change in anterior chamber depth following combined pars plana vitrectomy, phacoemulsification, and intraocular lens implantation using different types of intraocular lenses. Jpn J Ophthalmol.

[13] Yoshikawa Y, Matsushima T, Takano S, Makita J, Shinoda L (2024). Intraocular lens position stability during phacovitrectomy with air or gas tamponade (PREPRINT). Research Square.

[14] Borkenstein AF, Borkenstein EM (2022). Efficacy of large optic intraocular lenses in myopic eyes with posterior segment pathology. Ophthalmol Ther.

[15] Schrecker J, Seitz B, Langenbucher A (2022). Performance of a new 7 mm intraocular lens with follow-up over 1.5 years (Article in German). Ophthalmologe.

[16] Jeon S, Choi A, Kwon H (2022). Clinical outcomes after implantation of extended depth-of-focus AcrySof® Vivity® intraocular lens in eyes with low-grade epiretinal membrane. Graefes Arch Clin Exp Ophthalmol.

[17] Kim H, Jeon S (2022). Visual outcomes of epiretinal membrane removal after diffractive-type multifocal intraocular lens implantation. BMC Ophthalmol.

[18] Liu BS, Cui WN, Niu R, Chen Q, Nie ZT, Wei JT, Hu BJ (2021). Refractive outcomes after vitrectomy combined with phacoemulsification of idiopathic macular holes. Int J Ophthalmol.

[19] Hwang HS, Jee D (2011). Effects of the intraocular lens type on refractive error following phacovitrectomy with gas tamponade. Curr Eye Res.

[20] Ahfat FG, Yuen CH, Groenewald CP (2003). Phacoemulsification and intraocular lens implantation following pars plana vitrectomy: a prospective study. Eye (Lond).

